# Global monsoon precipitation responses to large volcanic eruptions

**DOI:** 10.1038/srep24331

**Published:** 2016-04-11

**Authors:** Fei Liu, Jing Chai, Bin Wang, Jian Liu, Xiao Zhang, Zhiyuan Wang

**Affiliations:** 1 Earth System Modeling Center and Climate Dynamics Research Center, Nanjing University of Information Science and Technology, China; 2 Department of Atmospheric Sciences and Atmosphere-Ocean Research Center, University of Hawaii at Manoa, Honolulu, HI 96825, USA; 3 Key Laboratory of Virtual Geographic Environment of Ministry of Education, School of Geography Science, Nanjing Normal University, Nanjing 210023, China

## Abstract

Climate variation of global monsoon (GM) precipitation involves both internal feedback and external forcing. Here, we focus on strong volcanic forcing since large eruptions are known to be a dominant mechanism in natural climate change. It is not known whether large volcanoes erupted at different latitudes have distinctive effects on the monsoon in the Northern Hemisphere (NH) and the Southern Hemisphere (SH). We address this issue using a 1500-year volcanic sensitivity simulation by the Community Earth System Model version 1.0 (CESM1). Volcanoes are classified into three types based on their meridional aerosol distributions: NH volcanoes, SH volcanoes and equatorial volcanoes. Using the model simulation, we discover that the GM precipitation in one hemisphere is enhanced significantly by the remote volcanic forcing occurring in the other hemisphere. This remote volcanic forcing-induced intensification is mainly through circulation change rather than moisture content change. In addition, the NH volcanic eruptions are more efficient in reducing the NH monsoon precipitation than the equatorial ones, and so do the SH eruptions in weakening the SH monsoon, because the equatorial eruptions, despite reducing moisture content, have weaker effects in weakening the off-equatorial monsoon circulation than the subtropical-extratropical volcanoes do.

Prediction of global monsoon (GM) change is important for infrastructure planning, food security, ecosystem services and sustainable economic development. The GM is defined by seasonal variation of precipitation^1^, and the diabatic heating associated with the monsoon precipitation generates a global-scale atmospheric overturning that varies according to different seasons^2^,^3^. The GM is essentially a response of coupled atmosphere-land-ocean system to seasonal cycle of solar forcing^4^. As shown in Fig. 1, the GM includes the Northern Hemisphere monsoon (NH monsoon) and the Southern Hemisphere monsoon (SH monsoon)^5^. The SH monsoon is composed of the South African, Australian and South American monsoons. The NH monsoon includes the South Asian, East Asian-western North Pacific, West African, and North American monsoons, which encompasses about 60% of the population on the planet and phenomenologically has profound effects on global climate^6^. Current studies on seasonal GM precipitation change involve both its internal variability and external forcing. The latter can be both natural (e.g., solar and volcanic activity) and anthropogenic (e.g., greenhouse gases, aerosols and land use)^6^,^7^,^8^.

This study is concerned with the responses of GM precipitation to volcanic eruptions, since large explosive volcanism is known to be a leading forcing of natural climate change on the interannual timescale. The most important mechanism by which a volcano perturbs climate is the injection of large amounts of SO_2_ gas into the stratosphere^9^. Such SO_2_ gas reacts with OH and H_2_O to form H_2_SO_4_ + H_2_O aerosols. The resulted aerosols interact with the incoming and outgoing radiation, which tends to perturb radiative balance and results in stratospheric warming and tropospheric cooling. Large volcanos can produce global cooling at the surface for typically 2 to 3 years^9^,^10^,^11^,^12^,^13^,^14^. Moreover, the slowdown of the water cycle is due to reduction of net surface shortwave radiation. The monsoon precipitation and the global mean precipitation are both found to decrease following large eruptions in the next few years^15^,^16^,^17^,^18^.

At present, it is unclear how the GM, as well as its two components, the NH monsoon and the SH monsoon, responds differently to large volcanic eruptions. Our results based on the instrumental data will show that the observed GM precipitation and the NH monsoon precipitation are reduced in a few years after large eruptions, while the SH monsoon precipitation on the other hand is enhanced. To figure out the reason of these different influences by volcanoes, it is worth explaining the different responses of the GM, the NH monsoon and the SH monsoon to large volcanic eruptions.

It is also not clear how the GM system responds to different volcanoes in terms of their eruption latitudes. The aerosols of historical volcanic eruptions have different meridional distributions^19^. What’s more, the equatorial and subtropical-extratropical volcanoes were found to have different effects on the tropical systems^20^. Recent studies suggested that sporadic volcanic eruptions in the NH strongly influence the surface temperature gradient and cause Sahelian drought, while large asymmetric stratospheric aerosol loadings concentrated in the SH induce a greening of the Sahel^21^. For the long-term impact, the equatorial eruptions were found to have greater impacts than the high-latitude eruptions on global climate because their stratospheric aerosol clouds cover a larger surface area and have a longer residence time, and because the aerosols are then transported poleward in both hemispheres and eventually cover the entire globe^16^.

We use climate model simulations to study these different responses of the GM precipitation to large volcanic eruptions. Estimating the role of explosive volcanism requires a large number of climate proxy data with good spatial distribution, while the number of suitable records decreases going back in time and large uncertainty also remains in the estimations of climate variability and forcing factors, especially before ~1600 AD^16^. To address the responses of monsoon precipitation to volcanic eruptions, coupled climate system models are particularly useful tools, since these climate models have been successfully employed to simulate the major observed effects of large volcanic eruptions^22^,^23^,^24^,^25^,^26^. Thus, in this study we will apply a state-of-the-art climate model to study the role of explosive volcanoes erupted at different latitudes in terms of their impacts on the GM precipitation.

## Stratification of volcanic forcing

Figure 2 shows the total aerosols and meridional distributions for each strong eruption with annual-mean global aerosol amount above 4 Tg (which is the averaged strength during 501–2000 AD) in the eruption year. There are 54 large explosive volcanoes during 501–2000 AD in total, and the strongest one is the Samalas volcano in 1257–1258 (Fig. 2a), which is followed by three smaller eruptions in 1268, 1275 and 1284. These strong volcanoes do not allow the climate to recover, and might have triggered the Little Ice Age^27^. Since the volcanic forcing data is based on Ice-core Volcanic Index 2 (IVI2), the strong volcano El Chichón at 1982, which is added in IVI2 version 2, is not included in IVI2.

We classify the volcanoes into three types based on the dataset in Gao *et al*.^19^, because aerosols from different volcanoes have distinctive meridional distributions. The first type is the NH volcanoes. Aerosols associated with this type have maximum column density in the NH and are barely transported to the SH. As for the SH volcanoes, on the other hand, the aerosols are mostly constrained in the SH and do not contribute much to the NH aerosol concentration. The last type is the equatorial volcanoes and their aerosols have maximum near the equator and decay poleward quickly. As shown in Fig. 2b, the eruption is called ‘the NH event’ when its aerosol column density is zero at 40°S, ‘the SH event’ when the aerosol column density is zero at 40°N, and ‘the equatorial event’ when the aerosol column density is above zero at both 40°S and 40°N. Based on this classification, the 54 eruptions during 501–2000 AD include 16 NH, 25 equatorial and 13 SH volcanic eruptions.

Each of the volcanic aerosols injected into the lower stratosphere by large eruptions only lasts 1–2 years and decays quickly thereafter. Figure 2c shows the aerosol evolution before and after each eruption in the dataset of Gao *et al*.^19^. Before the eruptions, the aerosol column density is zero. Afterwards, it increases quickly and reaches its maximum at the fifth month. After the eruptions, the aerosol concentration decays quickly, which has only half of the original amplitude in the second year and nearly recovers to the background state without new volcanoes in the third year following each eruption. Although volcanic aerosols are detectable after two years in many cases, in the data set of Gao *et al*.^19^, the aerosols of each eruption only stay in the stratosphere for about two years.

Since the volcanoes erupt in different months and have maximum aerosol column density in the fifth month after the eruption (Fig. 2c), we assume that the volcanos erupting in the monsoon season have negligible effect on the current monsoon system itself. For example, the eruptions beginning in the boreal summer do not affect the boreal summer NH monsoon in that year, and the boreal winter SH monsoon in that year and the boreal summer NH monsoon in the following year are said to occur in the “first year” after the eruptions. Thus, a simple way is used to define the “first year”: when the eruption happened before May, the 12 months starting from May of that year onward are defined as the “first year” for the NH and SH monsoons; when the eruption occurred after May while before October, the 12 months starting from November of that year onward are defined as the “first year”; when the volcano erupted after October, the 12 months starting from May of the next year onward is defined as the “first year”. The sum of the NH and the SH monsoon precipitation in the defined “first year” is used for the GM precipitation in “first year”.

Since the millennium simulation is forced by the volcanic forcing based on the reconstruction of Gao *et al*.^19^, this volcano dataset is also used to study the observed monsoon responses; in this way, the volcano stratification can be consistent in both simulation and observation analyses. There are some uncertainties in the hemispheric distribution and eruption dates in the reconstruction of Gao *et al*.^19^,^28^,^29^. These uncertainties, however, do not change the conclusion obtained from the sensitivity experiment.

## Observed GM precipitation responses to explosive volcanism

The long-term reconstructions of June-August monsoon Asian Palmer Drought Severity Index (PDSI) (Fig. 3a) and May-September Asian precipitation (Fig. 3e) give us a chance to look at how the strong Asian monsoon responds to different volcanic eruptions. The PDSI from 1300–2000 AD is reconstructed by Cook *et al*.^30^, and negative PDSI values indicate dry conditions, while positive values indicate wet conditions. The warm season (May-September) Asian precipitation from 1470–1999 is reconstructed by Feng *et al*.^31^.

Figure 3b–d show the superposed epoch analysis (Method) of monsoon Asian PDSI before and after each eruption of the NH, the SH, and the equatorial volcanoes. The normalization is applied in the superposed epoch analysis, thus all the following composites have no units. Figure 3b,c show opposite responses of the monsoon Asian PDSI to the NH and the SH eruptions. The monsoon Asian averaged PDSI is reduced significantly in the second year after the NH eruptions (Fig. 3b), while it is enhanced significantly in the first year after the SH eruptions (Fig. 3c). The equatorial eruptions tend to reduce the monsoon Asian PDSI significantly in both the first and second years after the eruptions (Fig. 3d). These results mean that the Asian monsoon is enhanced by the SH eruptions, while it is weakened by the NH and the equatorial eruptions.

The opposite responses of Asian monsoon to the NH and the SH eruptions are also clearly shown in the reconstructions of precipitation of Feng *et al*.^31^. The Asian monsoon precipitation is reduced after the NH eruptions, and the results are not significant (Fig. 3f), while the Asian monsoon precipitation is enhanced significantly after the SH eruptions (Fig. 3g). The equatorial eruptions also tend to reduce the Asian monsoon precipitation significantly (Fig. 3h).

**Figure 1:**
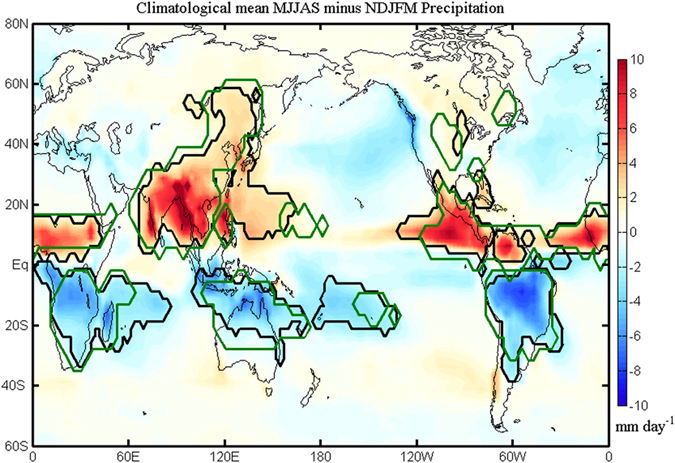
GM precipitation domain and annual reversal of precipitation. Shading shows the climatological mean MJJAS-minus-NDJFM precipitation using the GPCP data. The observed GM precipitation domains and the counterparts simulated in the 1500-year run are denoted by thick black and green lines, respectively. The GM precipitation domains outlined by the thick lines are defined by local summer-minus-winter precipitation rate exceeding 2.0 mm/d and the local summer precipitation exceeding 55% of the annual total. This figure was made using Matlab 2012b (http://www.mathworks.com/).

**Figure 2:**
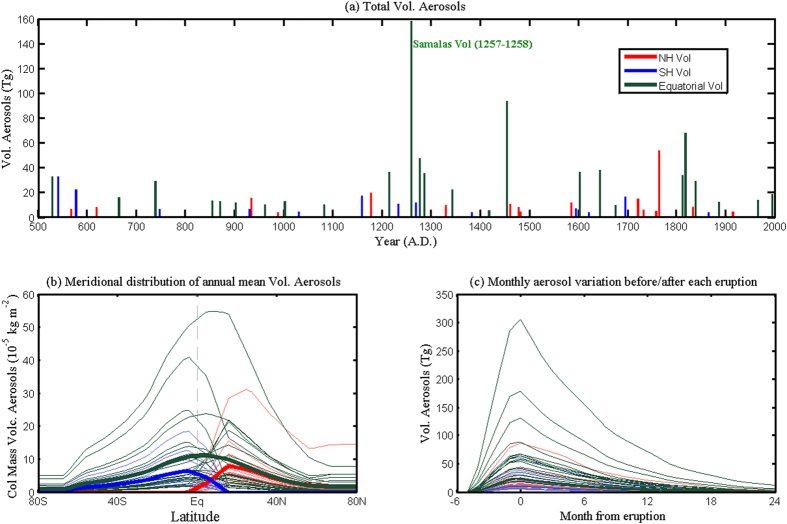
Volcanic aerosol forcing for the years 501–2000 AD. (**a**) Global average, annual-mean volcanic aerosols for 54 largest eruptions with annual aerosol injection above 4 Tg in the eruption year. Red color denotes the NH eruptions (16 times); blue, the SH eruptions (13 times); and dark green, the equatorial eruptions (25 times). (**b**) Meridional distribution of zonally-averaged, annual-mean aerosols column density for these 54 largest eruptions. The thick red, blue and dark green curves denote the averaged aerosol profiles for the NH, SH, and equatorial eruptions, respectively. (**c**) Monthly global average of volcanic aerosol injection before and after the eruptions for these 54 largest eruptions. Zero in the x-axis denotes the month when the aerosol injection is maximum. This figure was made using Matlab 2012b (http://www.mathworks.com/).

**Figure 3:**
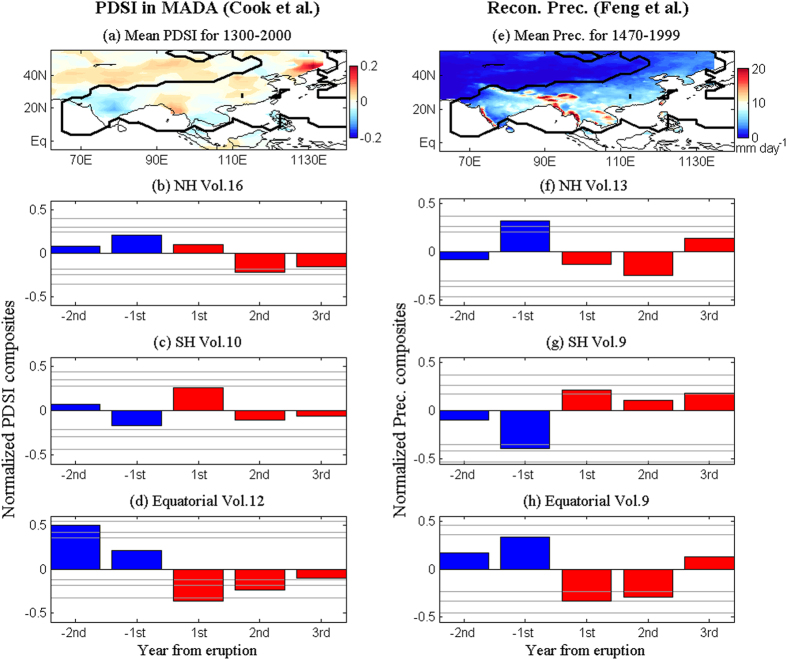
Long-term observed Asian summer monsoon responses to volcanic forcing. (**a**) Shading shows the mean PDSI for the period of 1300–2000 AD in the MADA reconstructed by Cook *et al*. The Asian monsoon precipitation domain outlined by the thick black line is the same as that in Fig. 1. Also shown are the superposed epoch analysis of normalized PDSI averaged in the Asian monsoon in responses to (**b**) 16 NH, (**c**) 10 SH and (**d**) 12 equatorial volcanic eruptions during 1300–2000 AD. Confidence limits (90%, 95%, 99%) are marked by horizontal lines. Red and blue colors mark the post-eruption and pre-eruption composites, respectively. “−1^st^” and “1^st^” in the x-axis denote the first year before and after the eruptions, respectively. Right panels are the same as in left panels but for the Asian precipitation during 1470–1999 reconstructed by Feng *et al*. In this period of 1470–1999 there have 13 NH, 9 SH, and 9 equatorial eruptions defined. This figure was made using Matlab 2012b (http://www.mathworks.com/).

**Figure 4:**
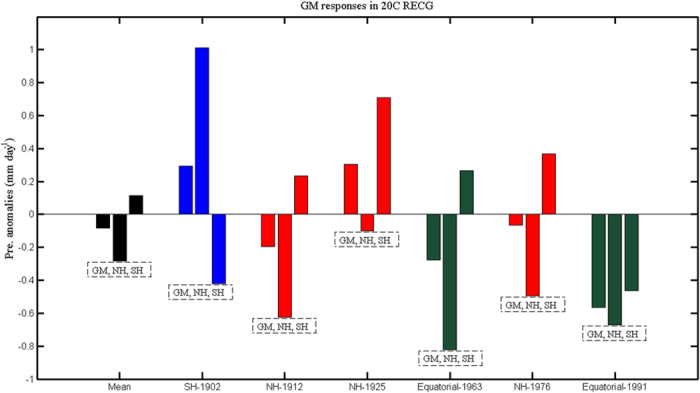
Instrumental monsoon responses. Difference of 2-year-mean monsoon precipitation between post- and pre-eruptions of the six large instrumental volcanoes (three NH eruptions in red, two equatorial eruptions in dark green, and one SH eruption in blue). The average responses to these six eruptions are also shown by the black bars. The three bars in each group denote the GM, NH monsoon, and SH monsoon precipitation, respectively. This figure was made using Matlab 2012b (http://www.mathworks.com/).

The reconstructed PDSI mainly represents the precipitation over the land, while the monsoon precipitation over the ocean cannot be estimated (Figs 1 and 3a). The other long-term reconstructions, such as the North American PDSI^32^ and the South-Eastern Australian rainfall^33^, can hardly capture the variability of the North American monsoon and the Australian monsoon that have large ocean components (Fig. 1); thus, we further look at the instrumental observation of the GM. We calculate the monsoon precipitation intensity by averaging the reconstructed precipitation anomalies from 20C RECG over the monsoon precipitation domain (black line in Fig. 1) defined by the GPCP precipitation (Method). For the instrumental period of 1900–2000, there are six large volcanic eruptions with their annual mean global total sulphate aerosol injection above 2 Tg (Method). Figure 4 shows that the difference between the 2-year-mean precipitation rate after and before the six large instrumental volcanoes is −0.08 mm day^−1^ when averaged over the GM regions; the difference is −0.28 mm day^−1^ over the NH monsoon regions, and is 0.12 mm day^−1^ over the SH monsoon regions. The GM and the NH monsoon are reduced by volcanic eruptions, while the SH monsoon is enhanced. In Fig. 4, the three NH eruptions are all found to reduce the NH monsoon precipitation, while they enhance the SH monsoon. Different from the NH eruptions, the SH eruption (only one case) has the opposite effect: it enhances the NH monsoon while weakening the SH monsoon. The two equatorial eruptions tend to reduce the NH monsoon precipitation, while one eruption enhances the SH monsoon and the other reduces the SH monsoon precipitation. To understand the different responses of the GM, the NH monsoon, and the SH monsoon to large volcanic eruptions at different latitudes, we use a numerical sensitivity experiment by the CESM1.

## Simulated GM precipitation responses to explosive volcanism

Under the volcanic forcing, CESM1 simulates a fairly realistic GM precipitation domain. Figure 1 also shows the climatological GM precipitation domain averaged for the whole 1500 years in this volcanic run. The NH monsoon domain (South Asian, East Asian-western North Pacific, West Africa, and North American monsoons) and the SH monsoon domain (South African, Australian and South American monsoons) are well captured in this simulation, except for the western North Pacific monsoon and eastern part of the Australian monsoon domains, which are too small compared to the observation. The monsoon precipitation intensity is calculated by the precipitation averaged over the simulated monsoon domain.

**Figure 5:**
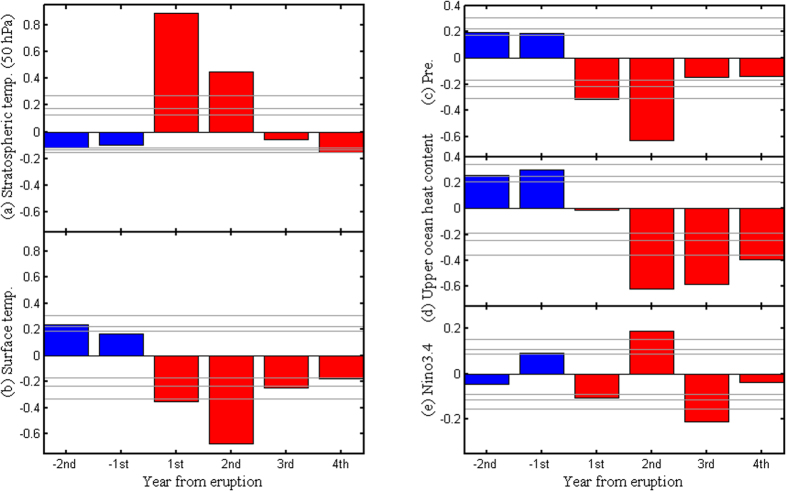
Simulated superposed epoch analysis of normalized model responses to large volcanic eruptions. Global mean (**a**) stratospheric temperature (50 hPa), (**b**) surface temperature, (**c**) precipitation, (**d**) upper ocean heat content (average temperature from 0–300 m), and (**e**) Niño 3.4 index defined by the area (120°W–170°W, 5°S–5°N) averaged sea surface temperature anomaly, in response to 54 large volcanic eruptions during 501–2000 AD. Confidence limits (90%, 95%, 99%) are marked by horizontal lines. Blue and red colors mark the pre-eruption and post-eruption composites, respectively. “−1^st^” and “1^st^” in the x-axis denote the first year before and after the eruptions, respectively. This figure was made using Matlab 2012b (http://www.mathworks.com/).

Before analyzing the GM response, we first examine how good this model responds to large volcanic eruptions. Figure 5 shows the responses of global-mean stratospheric temperature, surface temperature, precipitation, upper-ocean heat content, as well as the Niño 3.4 index. In a few years after the eruptions, the aerosols in the stratosphere result in stratospheric warming (Fig. 5a) and surface cooling (Fig. 5b), which are in agreement with previous works^9^,^10^,^11^,^12^,^13^,^14^. Consistent with the finding of slowing down of the water cycle due to the reduction in net surface shortwave radiation^15^,^16^,^17^,^18^, the global precipitation is also reduced by the volcanic forcing (Fig. 5c). Although the global ocean becomes cooler (Fig. 5d), the El Niño-like sea surface temperature (SST) anomaly, represented by positive Niño 3.4 index, is reproduced in the second year after the eruptions (Fig. 5e), which agrees with previous works that show large volcanic eruptions tend to increase the occurrence of El Niño^20^,^34^,^35^,^36^. The above results show that this volcanic sensitivity experiment can simulate reasonable responses to large volcanic forcing.

**Figure 6:**
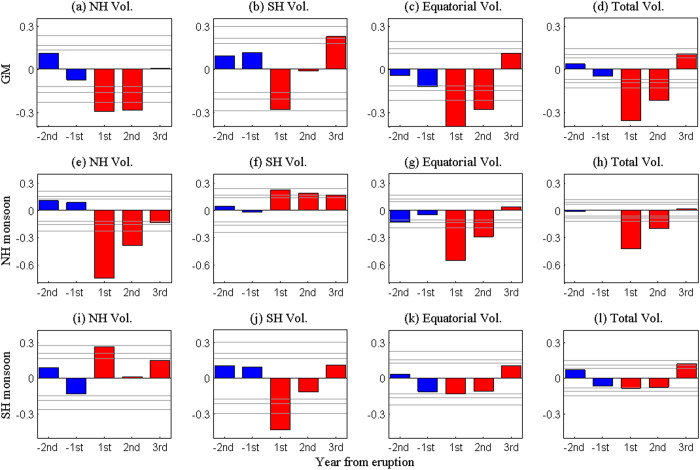
Simulated superposed epoch analysis of normalized monsoon responses to different large volcanic eruptions. The GM precipitation intensity in responses to (**a**) NH, (**b**) SH, (**c**) equatorial, and (**d**) total volcanic eruptions. The responses of NH monsoon and SH monsoon precipitation are shown in the middle (**e**–**h**) and bottom (**i**–**l**) panels. Confidence limits (90%, 95%, 99%) are marked by horizontal lines. Blue and red colors mark the pre- and post-eruption composites, respectively. “−1^st^” and “1^st^” in the x-axis denote the first year before and after the eruptions, respectively. This figure was made using Matlab 2012b (http://www.mathworks.com/).

The responses of GM precipitation intensity to different types of volcanic eruptions can be explored by the superposed epoch analysis. Figure 6a–d show the composite GM precipitation intensity before and after the NH, the SH, the equatorial, and total volcanoes. The GM precipitation is significantly (above the 99% confidence level) reduced in the two years following each eruption of the NH volcanoes (Fig. 6a) and the equatorial volcanoes (Fig. 6c). The SH eruptions also result in a significant (above the 95% confidence level) reduction of the GM precipitation in the first year and the GM is less affected in the second year (Fig. 6b). To sum up, the GM precipitation is reduced by the volcanic eruptions in the following 1–2 years after each eruption (Fig. 6d). Also of interest is the rebound of GM precipitation in the third year after the eruptions. If we assume that all volcanos have the same strength as the last strong one, the Pinatubo at 1991, the total volcanos will cause a reduction of 0.09 mm day^−1^ for the GM precipitation in the first year (Fig. 6d), which has the same order of magnitude as the observation (Fig. 4).

To further understand the role of explosive volcanism, the subsystems of the GM are studied. Figure 6e through Fig. 6h show the responses of NH monsoon precipitation to different eruption groups. After the eruptions of NH volcanoes, the NH monsoon precipitation is remarkably reduced in the following two years, and the maximum reduction occurs in the first year, implying severe droughts (Fig. 6e). The equatorial eruptions also have a drying effect on the NH monsoon (Fig. 6g), but the magnitude of reduction is smaller than that induced by the NH eruptions. The SH eruptions, in contrast, tend to significantly enhance the NH monsoon precipitation in the three years following the eruptions (Fig. 6f). The total effect of all eruptions is to reduce the NH monsoon precipitation (Fig. 6h). These results imply that the NH monsoon precipitation is reduced by the large NH and equatorial eruptions, and that the NH eruptions have a stronger effect than the equatorial ones. The NH monsoon precipitation is enhanced by the SH eruptions. The explanation for these divergent effects of different types of eruptions will be given in the next section.

The responses of SH monsoon precipitation to three types of eruptions are different from those of NH monsoon precipitation, which are shown in Fig. 6i–l. The SH monsoon precipitation is significantly enhanced by the NH volcanoes in the first year, and a weak increase can be seen in the second and third years (Fig. 6i). The SH eruptions tend to reduce the SH monsoon precipitation profoundly in the first year, and this reduction is not significant in the second year (Fig. 6j). The equatorial and total eruptions, however, have drying effect on the SH monsoon precipitation, and this negative effect is significant in the first year (Fig. 6k,l). There are evident asymmetric effects of the NH and the SH eruptions on the SH monsoon precipitation. The SH eruptions have a stronger effect on reducing the SH monsoon precipitation than the equatorial eruptions do, and the NH eruptions enhance the SH monsoon precipitation. For a lower threshold of 2 Tg, similar results are obtained (Figure not shown). More sensitivity experiments have also been done to show that different thresholds for defining the “large eruption” do not change the results qualitatively, except that the results with high thresholds are more significant than those with low ones.

## Dynamics of the asymmetric volcanic forcing

**Figure 7:**
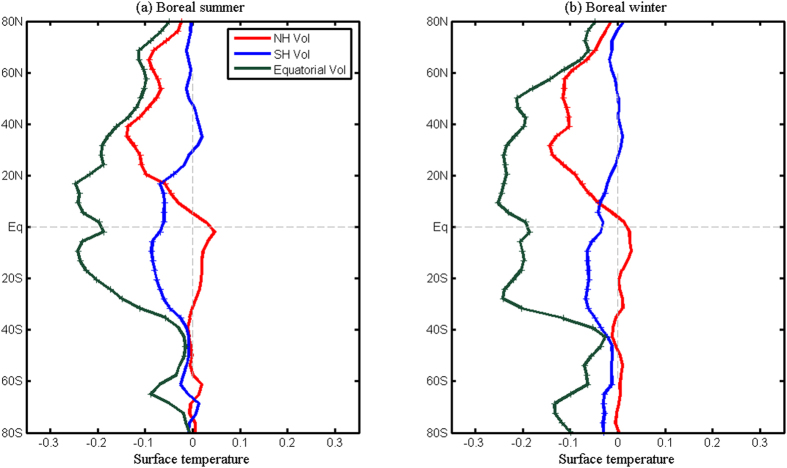
Simulated superposed epoch analysis of normalized surface air temperature responses to different explosive volcanoes. (**a**) Anomaly composite of zonal mean surface air temperature averaged in the first two boreal summers following the eruptions of the NH (red), SH (blue) and equatorial (dark green) volcanoes. Statistically significant (95% one-tailed) epochal anomalies based on Monte Carlo resampling (n = 10,000) are indicated by crosses. (**b**) Same as in (**a**), except for the boreal winter. This figure was made using Matlab 2012b (http://www.mathworks.com/).

Different meridional distributions of aerosols contribute to the aforementioned different responses of NH monsoon and SH monsoon. In order to understand the asymmetric responses of these monsoon systems, we show in Fig. 7 the changes of zonally-averaged surface air temperature (hereafter surface temperature) induced by these three types of eruptions for the boreal summer and winter, respectively. In the boreal summer (Fig. 7a), the NH eruptions reduce the surface temperature in the NH, and the maximum cooling occurs around 30°N–40°N. The SH, however, is less affected by the NH volcanoes, and hence southward gradient of temperature in the NH subtropical region is caused by the NH volcanoes. The cooling induced by the SH eruptions span tropical and SH regions, and the mid-latitude of the NH is less affected; thus, the strongest northward gradient of temperature in the NH subtropics is induced by the SH eruptions. The equatorial eruptions, however, tend to cool the tropical and subtropical regions in both hemispheres. In boreal winter (Fig. 7b), the results are similar as in boreal summer. In summary, the NH volcanoes primarily cool the NH while keeping the SH temperature less affected. The SH volcanoes cool the SH and the tropics, while weakly affecting the NH. Both NH and SH volcanoes create hemispheric temperature gradient anomalies. The equatorial volcanoes tend to cool both hemispheres strongly.

**Figure 8:**
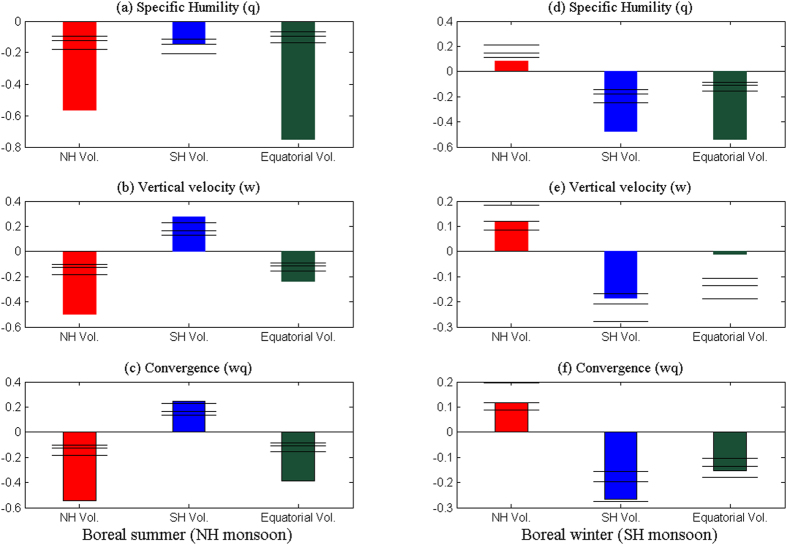
Simulated superposed epoch analysis of normalized moisture convergence responses to different explosive volcanoes. Anomaly composites of NH monsoon mean of (**a**) specific humility at 1000 hPa, (**b**) vertical velocity at 500 hPa, and (**c**) moisture convergence averaged in the first two boreal summers following the eruptions of the NH (red), SH (blue) and equatorial (dark green) volcanoes. Right panels denote the responses for the SH monsoon in the boreal winter (**d**–**f**). Confidence limits (90%, 95%, 99%) are marked by horizontal lines. This figure was made using Matlab 2012b (http://www.mathworks.com/).

These different distributions of surface temperature anomalies cause different moisture convergence patterns that induce large-scale precipitation changes of the GM. Figure 8 shows the convergence and moisture change induced by different types of volcanoes. In boreal summer (Fig. 8a–c), the NH eruptions induce negative moisture anomalies and strong divergence (or downward motion) anomalies in the NH monsoon, which result in strong moisture divergence anomalies in the NH monsoon. The SH eruptions, although reducing the moisture content, produce anomalous ascent motions and moisture convergence anomalies in the NH monsoon. The equatorial eruptions excite very weak convergence anomalies, while they reduce moisture heavily; thus, negative moisture convergence anomalies are induced in the NH monsoon.

In the boreal winter (Fig. 8d–f), the opposite results are seen. The NH eruptions induce positive moisture convergence in the SH monsoon through exciting positive convergence anomalies. The SH eruptions induce strong moisture divergence in the SH monsoon since the SH eruptions reduce moisture and induce strong divergence. The equatorial eruptions also induce moisture divergence through reducing moisture heavily.

These results explain why the NH eruptions are more efficient in reducing the NH monsoon precipitation than the equatorial eruptions (Fig. 8a–c). Although the equatorial eruptions cool the globe and reduce the moisture most heavily, they only excite very weak divergence anomalies in the NH monsoon region; thus, only weak moisture divergence anomalies are produced. After the eruptions of NH volcanoes, the moisture is much reduced in the NH monsoon, and strong divergence anomalies are also excited there. Both of these two negative effects tend to reduce the NH monsoon precipitation heavily. Thus, the NH eruptions have a stronger drought effect on the NH monsoon than the equatorial eruptions do. In the same way, the SH eruptions also reduce the SH monsoon precipitation more strongly than the equatorial eruptions do (Fig. 8d–f).

The NH monsoon precipitation is increased by the SH volcanoes and the SH monsoon is also enhanced by the NH eruption. The analysis shows that this increase is caused by the enhanced circulation and associated positive convergence anomalies rather than moisture increase (Fig. 8). This is different from the monsoon intensification induced by anthropogenic greenhouse gases. Under the global warming induced by strong greenhouse-gas concentration, the GM precipitation is enhanced by the moistened atmosphere, while the circulation is weakened^37^.

## Summary and Discussion

Although the explosive volcanoes tend to reduce GM precipitation in a few years after the eruptions (Fig. 6a–d), the NH monsoon and the SH monsoon have different responses to extratropical explosive volcanoes because of the asymmetric aerosol distributions. The NH eruptions are found to be more efficient in reducing the NH monsoon precipitation than the equatorial eruptions (Fig. 6e,g), and the SH eruptions are also more efficient in reducing the SH monsoon precipitation than the equatorial eruptions (Fig. 6j,k), because the extratropical eruptions tend to reduce moisture heavily and cause strong divergence anomalies, while the equatorial volcanoes only act to reduce the moisture and do not affect the circulation field much (Fig. 8).

The extratropical volcanism also reveals a new process to affect the NH monsoon or the SH monsoon precipitation. Different from the GM precipitation intensification induced by increasing moisture associated with the global warming caused by strong greenhouse-gas concentration^37^, the SH eruptions produce asymmetry temperature anomalies characterized by strong SH cooling and weak NH temperature change (Fig. 7a); thus, the enhanced hemispheric thermal contrast generates meridional pressure gradient that drives low-level cross-equatorial flows from the SH, which converges into the monsoon trough regions in the NH. These convergence anomalies induced by the SH eruptions tend to enhance the NH monsoon precipitation dramatically (Fig. 8a–c). The NH volcanoes also enhance the SH monsoon precipitation in a similar way (Fig. 8d–f).

Based on the long-term reconstruction of PDSI and precipitation, the Asian monsoon in the NH is also found to be enhanced by the SH eruptions, while it is weakened by the NH and the equatorial eruptions (Fig. 3). Since 1900, there have been three large NH volcanoes, two equatorial volcanoes and one SH volcano^19^. Thus, the more frequently erupted NH volcanoes tend to reduce the NH monsoon precipitation more heavily, while they enhance the SH monsoon precipitation (Fig. 4). Our new finding shows that the GM also changes significantly due to the volcanic forcing, and the three types of volcanoes, namely, the NH, the SH and the equatorial volcanoes, have different effects on the NH monsoon and the SH monsoon. These results are based on the superposed epoch analysis that has been widely used to study the climate responses to large volcanic eruptions^34^,^38^,^39^. We agree that the internal variability plays an important role in GM variation^6^,^40^.

The El Niño-Southern Oscillation (ENSO) also exerts a predominant influence on the interannual variation of the GM and its different components^6^,^40^,^41^,^42^. In recent studies, a large volcanic eruption was found to increase the occurrence of El Niño in the observations^20^,^34^, and in the simulations^35^,^36^. Our results also confirm that the El Niño events can be generated following the large volcanic eruptions (Fig. 5e). In view of its complexity and importance, this issue deserves a further in-depth analysis. But such a detailed analysis is beyond the scope of the present work and will be reported elsewhere.

In this study, we mainly focus on the short-term impacts of explosive volcanism. Previous study also showed long-term effects of equatorial eruptions^16^, which is attributed to the cooling of high-latitude NH produced by multiple equatorial eruptions, since positive feedbacks associated with ice and snow cover can lead to long-term climate cooling in the Arctic. The natural variations composed of solar radiation and volcanic eruptions can also induce the millennium scale variation and the quasi-bicentennial oscillation in GM precipitation^7^,^8^. Since there is an increasing demand for decadal climate prediction, the responses of the GM to external volcanic forcing on the decadal timescale should be studied in the future.

## Methods

### Data sources

The monthly-mean volcanic forcing for the years 501–2000 AD, the Ice-core Volcanic Index (IVI2), is obtained from Gao *et al*.^19^, which is derived from 32 Arctic and 22 Antarctic ice-core records. The meridional distribution of aerosols injected into the low stratosphere is determined according to their eruption latitudes. This data is available at http://www1.ncdc.noaa.gov/pub/data/paleo/climate_forcing/volcanic_aerosols/gao2008ivi2. We use the monthly-mean precipitation data from the Global Precipitation Climatology Project (GPCP) data version 2.2 from 1979–2010 to define the GM domain in the observation^43^. To represent the long-term monsoon precipitation change, the reconstructed monthly mean precipitation anomalies from 1900–2008 are obtained from the merged statistical analysis of historical monthly-mean precipitation anomalies REConstructed Globally (hereafter 20C RECG) at a 2.5° spatial resolution^44^. The Monsoon Asia Drought Atlas (MADA), a seasonally resolved gridded spatial reconstruction of Asian monsoon drought and pluvials during 1300–2005 AD, is used to measure the Asian monsoon precipitation^30^. This data is available at http://www.ncdc.noaa.gov/paleo/pdsi.html. A gridded reconstruction of warm season (May-September) precipitation over the Asian continent (5°–55°N, 60°–135°E) back to 1470 is also used, which is on the basis of tree-ring data, historical documentary records, ice core records, and few long-term instrumental data series^31^.

### Definition of ‘large’ volcanic eruption

In this study, an eruption is considered as a ‘large’ one when its annual mean aerosol column density in the eruption year exceeds the mean volcanic aerosol strength. The mean volcanic aerosol strength is defined by the average of annual mean global total sulphate aerosol injection for all years when aerosol injection is not zero. Use of different critical values does not change the results qualitatively. For the whole study period from 501–2000 AD^19^, the mean volcanic aerosol strength is 4 Tg. During the period 1300–2000 for the MADA data, the period 1470–1999 for the reconstruction of Asian precipitation, and the period 1900–2000 for the 20C RECG data, the volcanic eruptions are weak and the mean volcanic aerosol strength is about 2 Tg. In order to include more volcanic events, we use a lower threshold of 2 Tg for these periods.

### Calculation of the GM domain

The simulated GM precipitation domain reflects a model’s capability for simulating the seasonal distribution of precipitation as well as the total amount of annual precipitation at each location^1^,^5^. As a dominant mode of the annual variation of tropical precipitation and low-level winds, the GM domains can be delineated by the regions where the annual range (local summer mean minus local winter mean) of precipitation rate exceeds a threshold of 2.0 mm/day and the local summer precipitation exceeds 55% of the annual total^7^,^45^,^46^. This metric has been shown to be very useful for gauging a climate model’s performance^4^,^6^,^46^,^47^. The NH monsoon precipitation intensity is defined by the boreal summer (May through September) precipitation averaged over the NH monsoon region, and the SH monsoon precipitation intensity is by the boreal winter (November through March) precipitation averaged over the SH monsoon area. The GM precipitation intensity is the sum of the two.

### Model introduction and experimental design

The model used in this study is the Community Earth System Model version 1.0 (CESM1) developed by the National Center for Atmospheric Research (NCAR)^48^. The CESM1 is a fully-coupled global Earth System Model. It is composed of five separate model components, simulating the Earth’s atmosphere, ocean, land, land ice, and sea ice linked by one central coupler. The CESM1 is based on the Community Climate System model (CCSM) with improved physics, parameterizations and additional biogeochemical cycle processes. Climate responses to large volcanic eruptions were well studied using an earlier version, the fully-coupled state-of-the-art NCAR Community Climate System Model version 3 (CCSM3)^16^.

Model resolutions used in this study are as follows: The horizontal resolution is approximately 3.75° × 3.75° in CAM4. The land model is the Community Land Model (CLM4), which adopts the same horizontal resolution as the CAM4. POP2 has 116 unevenly distributed grids in latitude and 100 grids in longitude. Twenty-six vertical levels in the Community Atmosphere Model (CAM4) and 60 vertical levels in the Parallel Ocean Program version 2 (POP2).

Based on a 2000-year control simulation, the same as those control experiments participating in the PMIP3 in which the external forcing is fixed to the year 1850^49^, a 1500-year forcing simulation is performed from 501 to 2000 AD, in which the only changing external forcing is the volcanic forcing obtained from the reconstructions of volcanic aerosols covering the period of 501–2000 AD^19^.

### Superposed epoch analysis

In this study, we use the superposed epoch analysis^50^ to evaluate the influence of explosive volcanoes on GM precipitation anomalies. A conventional bootstrapped resampling with replacement is used, and the confidence intervals are calculated by repeating the superposed epoch analysis using repeated (n = 10,000) random draws of pseudo–‘event’ year from the available time span. Significance is then evaluated by comparing percentiles from the random draw to the composite mean of the real data. Following Adams *et al*.^34^ and Anchukaitis *et al*.^39^, we normalize the data by the magnitude of the largest absolute anomaly magnitude in each window to avoid the possibility that any single eruption would dominate the epochal signal. Each window includes 15 years with 7 years before and 8 years after each eruption.

## Additional Information

**How to cite this article**: Liu, F. *et al*. Global monsoon precipitation responses to large volcanic eruptions. *Sci. Rep.* **6**, 24331; doi: 10.1038/srep24331 (2016).
